# False certainty in the acquisition of anatomical and physiotherapeutic knowledge

**DOI:** 10.1186/s12909-022-03820-x

**Published:** 2022-11-08

**Authors:** Johannes von Hoyer, Martina Bientzle, Ulrike Cress, Johannes Grosser, Joachim Kimmerle, Peter Holtz

**Affiliations:** 1 Knowledge Construction Lab. Leibniz-Institut Für Wissensmedien, Schleichstr. 6, 72076 Tuebingen, Germany; 2 Department of Applied Cognitive Psychology and Media Psychology, University of Tuebingen, Schleichstr.4, D-72070 Tuebingen, Germany, Tuebingen, Germany

**Keywords:** Medical Education, Testing/Assessment, Metacognition, False certainty

## Abstract

**Background:**

Efficient metacognitive monitoring, that is the monitoring of one’s own thought processes and specifically one’s state of knowledge, is essential for effective clinical reasoning. Knowing what one does and does not know is a competency that students of health professions need to develop. Students often tend to develop false certainty in their own knowledge in the longer course of their education, but the time frame that is required for this effect to occur has remained unclear. We investigated whether students developed false certainty already after one course unit.

**Methods:**

This study analysed data from one sample of medical students and four samples of physiotherapy students in two formal educational settings (total *N* = 255) who took knowledge tests before and after a course unit. We examined changes in students’ confidence separately for correctly and incorrectly answered questions and analysed their ability to assign higher levels of confidence to correct answers than to incorrect answers (discrimination ability).

**Results:**

Students’ knowledge as well as confidence in their correct answers in knowledge tests increased after learning. However, consistently for all samples, confidence in incorrect answers increased as well. Students’ discrimination ability improved only in two out of the five samples.

**Conclusions:**

Our results are in line with recent research on confidence increase of health professions students during education. Extending those findings, our study demonstrated that learning in two different formal educational settings increased confidence not only in correct but also in incorrect answers to knowledge questions already after just one learning session. Our findings highlight the importance of improving metacognition in the education of health professionals—especially their ability to know what they do not know.

**Supplementary Information:**

The online version contains supplementary material available at 10.1186/s12909-022-03820-x.

## Background

### Metacognitive monitoring

Efficient metacognition, that is, a range of cognitive processes that are involved in self-assessment, cognitive control, and monitoring, is paramount for effective clinical reasoning processes [1]. One major part of metacognition is metacognitive monitoring of one’s own knowledge, which allows an individual to gain insight into what they know and what they do not know [2]. However, practicing medical professionals can lack awareness of what they do and do not know [3–6].

The ability of metacognitive monitoring can be expressed by metacognitive calibration, that is, the association between knowledge and confidence in that knowledge [7]. In general, if individuals display more confidence than would be appropriate given their knowledge, they are poorly calibrated, and overconfidence occurs. Even experienced medical practitioners might not always be aware whether their diagnoses are correct or not [8] and physicians’ levels of confidence in their diagnoses sometimes show little correlation with their diagnostic accuracy [9]. Overconfidence of medical professionals is widespread: They overestimate what they know in areas like dermatology [10], pathology [11], radiology [12], internal medicine [13], and dementia [14]. Dermatologists, for example, change their minds about the malignancy of lesion images less often if they are more confident in their decisions [10], yet they follow this tendency regardless of whether their initial decision was correct or incorrect. Apart from its importance in professional life, adequate monitoring of what is known is also critical in medical education since it allows a learner to control and regulate one’s own learning processes [15, 16].

### Metacognitive judgments of confidence

One common method of measuring monitoring is collecting metacognitive judgments by asking students to rate confidence in the correctness of their answers to knowledge test items [17]. Adequate monitoring ability should be reflected by high confidence ratings to correctly and low confidence ratings to incorrectly answered questions [18]. Especially knowing what one does not know, is a competency that needs to be taught in medical and health professional education, since a health professional should sense when to ask for another opinion in making decisions [19].

The literature suggests that medical students show persistent overconfidence in diverse medical topics like delirium [15], surgery [20], or acne [21]. It should be expected that over a student’s years of study, overconfidence would give way to better monitoring ability. Yet the data of a recent longitudinal study by Kämmer et al. [17] points to the opposite direction. The authors measured students’ medical knowledge and confidence in knowledge questions asked over the course of 10 semesters. As a measure of metacognitive calibration, they analysed students’ discrimination ability, that is, the ability to assign higher levels of confidence to correct answers than to incorrect answers. The authors report an increase of medical knowledge over the course of the semesters but no change in the discrimination ability. Instead, results showed a general increase in the absolute level of confidence over time, regardless of knowledge.

The results of another recent longitudinal study by Cecilio-Fernandes and colleagues [22] found that students in their final year answered significantly more clinical knowledge questions correctly than first-year students. However, first-year students on average marked 71% of their incorrectly answered questions as “don’t know”, whereas final-year students did so only for 37%. Those results suggest that knowledge gain over the course of one’s medical education can be accompanied by an emerging reluctance to admit one’s lack of knowledge. Those results indicate that over the course of medical education, while students acquire medical knowledge and increase confidence in this knowledge, they also develop certainty in supposed knowledge they mistakenly think is correct. Due to the longitudinal nature of these studies, it is unclear what time frame it will take for this effect to emerge in health professions education. Thus, the goal of the study presented here was to explore how medical and physiotherapy students’ confidence in their knowledge as well as their metacognitive calibration changes after only one course unit. Specifically, we were interested in addressing the research question of whether students would develop false certainty under these conditions.

## Materials and method

We analysed five datasets containing five student samples to explore medical and physiotherapy students’ change in confidence before and after a course unit in two different formal medical educational settings. The design of all of the studies was similar in that it featured a learning phase and the pre-/post-measurement of both knowledge and confidence. Courses varied in content and didactic methods. Samples 1 and 2 were taken from a study published by Grosser and colleagues [23] and included medical and physiotherapy students who watched an anatomy lecture broadcasted live from the dissection hall of a university clinic’s anatomical institute. This live dissection was moderated by an anatomy professor. The authors did not report changes in confidence or metacognitive indices in the previous publication, which are the dependent variables in our analyses. Samples 3 to 5 were taken from three different cohorts of a physiotherapy course about gait analysis and included physiotherapy students. The data of the students in Samples 3 and 4 have not been published; the data of Sample 5 was partly published by Bientzle and colleagues [24].

### Participants

**Fig. 1:**
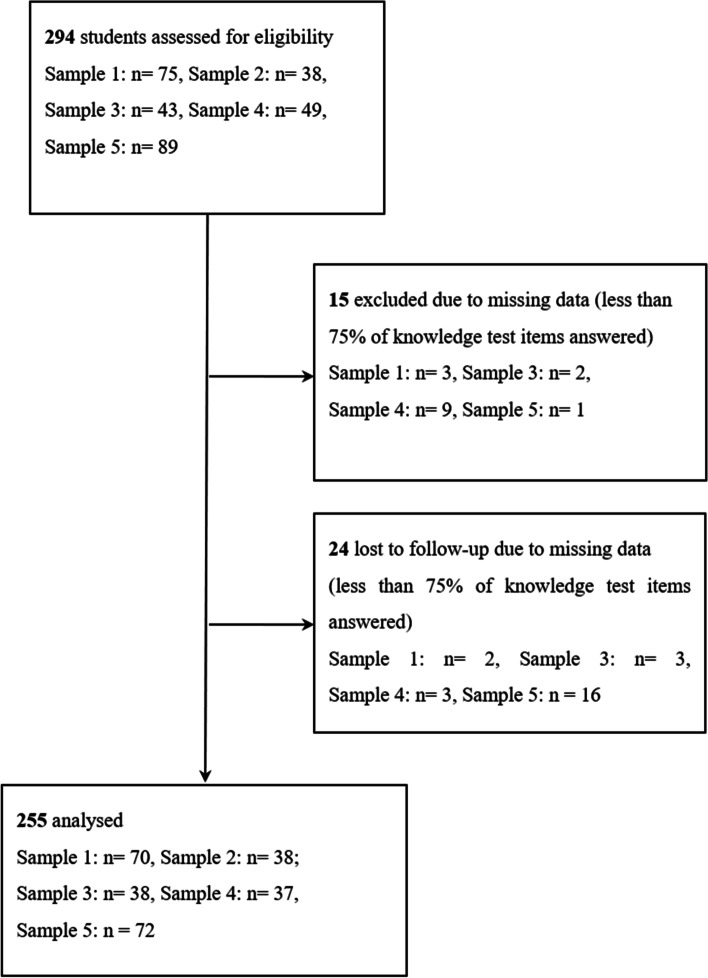
Flow chart of the data preparation procedure

Data of initially *N* = 294 participants was collected in four separate studies (Samples 1 and 2 were collected together; Samples 3–5 were collected individually in each case but using the same setting). Participants for Samples 1 and 2 were recruited among medical and physiotherapy students who participated in an educational live event about anatomy. Participants of Samples 3–5 were students of physiotherapy who participated in a course about gait analysis. Participants’ knowledge about the course content was tested before and immediately after the course (Samples 1, 2) or one week later (Samples 3–5). Participants who responded to fewer than 75% of knowledge test items or confidence judgments at either time 1 or time 2 were excluded from our analyses. See Table 1 for the samples’ characteristics and Fig. 1 for a flow diagram.

**Table 1:** Characteristics and demographics of samples analysed

Sample	Degree programme	N	Sex female/male/NA	Mean Age (SD)	Year of training
Sample 1	Medicine	70	48/22/-	21.57 (3.78)	Beginning of second year
Sample 2	Physiotherapy	38	22/10/-	21.92 (2.94)	Beginning of second (24) and third year (14)
Sample 3	Physiotherapy	38	25/12/1	19.95 (2.37)	First year
Sample 4	Physiotherapy	37	13/24/-	20.50 (2.16)	First year
Sample 5	Physiotherapy	72	55/15/1	21.74 (4.20)	First year

### Measures

#### Knowledge tests

All knowledge tests were forced choice tests in which participants were given one statement at a time and had to determine whether the statement was true or false. Samples 1 and 2 responded to a 24-item knowledge test about the anatomy of the shoulder. Samples 3–5 responded to a 20-item knowledge test on gait analysis. Presenting knowledge tests to students that require them to select the correct answer from multiple statements is a straightforward and standardized approach for assessing knowledge in medical education [25].

#### Confidence

Following each knowledge test item, students’ confidence was measured by asking them to indicate how confident they were that their given answer was true on a 6–point rating scale, with labelled endpoints of 1 = “not sure at all” and 6 = “absolutely sure” for every item of the knowledge tests.

### Statistical analyses

For answering the research question of whether false certainty would already develop after only one course, we analysed the change of confidence over time for each sample. To compare effect sizes between samples we first z-transformed the confidence variable. Then we conducted mixed effects model regressions separately for correct and incorrect answers for each sample. This method is able to handle the unbalanced cell size of our data [26]. We specified confidence as the dependent variable and time as a fixed effect. As random effects, we specified random intercepts for participant and item, as well as a by-participant random slope for time. We report standardized coefficients as well as confidence intervals for all models.

For analysing knowledge gain, for each participant we calculated the mean number of knowledge test items answered correctly at t1 and t2. We then used a paired sample t-test to analyse if there was an increase in knowledge at t2.

As measure of metacognitive calibration, we examined participants’ discrimination ability. Specifically, we used the index of *relative metacognitive accuracy* by calculating the Goodman–Kruskal’s gamma correlation [27] between the correctness of an answer and the respective confidence judgment. This is a widely endorsed method in educational psychology and metacognition research [28–32]. Individuals’ relative accuracy can range between -1 and 1, with 1 indicating perfect discrimination ability between correct and incorrect answers. For each participant we calculated the mean relative accuracy at both measurement points and determined changes in those scores by paired sample t-tests.

An alpha level of 0.05 was used for all statistical tests. Effect sizes for all mean differences are indicated as Cohen’s d; confidence intervals are also given. We used statistical software R version 4.0.3 (2020–10-10) for all analyses. Data and code can be found at https://osf.io/6v8za/?view_only=4a8fd507cceb42c1b8a8e9070d247728.

## Results

### Knowledge gain

**Fig. 2:**
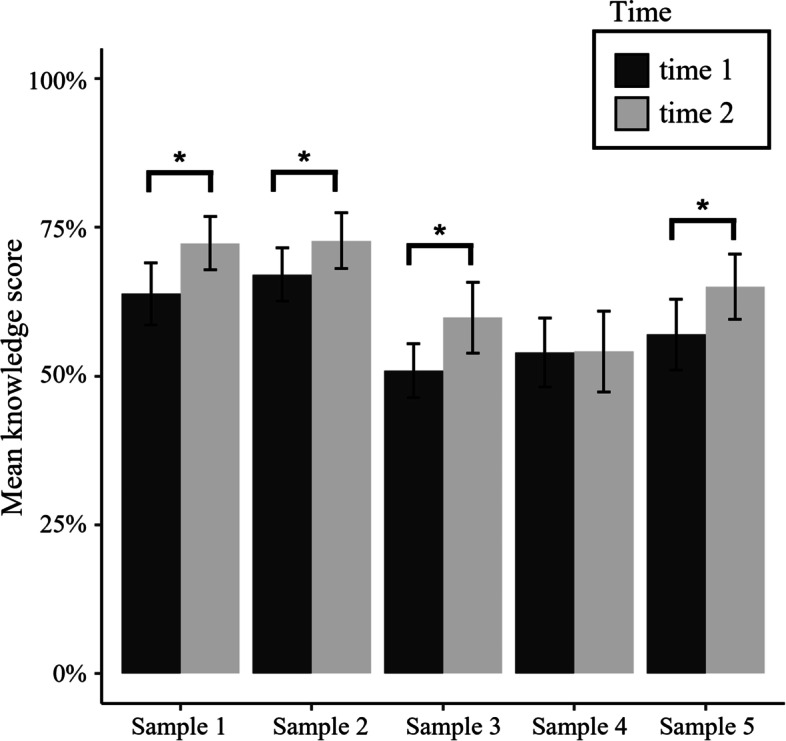
Mean percentage of correctly answered questions for samples and measurement point. Error bars represent standard deviations. Asterisks indicate significant differences as determined by paired sample t–tests

Students in Samples 1, 2, 3, and 5 increased their knowledge significantly as indicated by paired sample t-tests, whereas in Sample 4 we could not detect an increase in knowledge. The first section of Table 2 shows statistical parameters for those analyses including *p*-values. Figure 2 shows a bar graph depicting the mean percentage of correctly answered knowledge test items for all samples and both measurement points.

**Table 2:** Descriptives as well as pairwise comparisons for proportion of correctly answered questions and confidence, separated for correct and incorrect answers, and including effect sizes and their confidence intervals (CIs)

	**t1**	**t2**					
**Sample**	**M (SD)**	**M (SD)**	**t**	**df**	**p**	**Cohen’s_d**	**CIs**
*Relative number of correctly answered statements*							
Sample 1	0.64 (0.10)	0.72 (0.09)	6.85	69	< .001	0.87	0.58—1.17
Sample 2	0.67 (0.09)	0.73 (0.09)	3.83	37	< .001	0.62	0.27—0.97
Sample 3	0.51 (0.09)	0.60 (0.12)	3.81	36	< .001	0.84	0.33—1.34
Sample 4	0.54 (0.12)	0.54 (0.14)	0.08	36	.938	0.01	-0.31—0.33
Sample 5	0.57 (0.12)	0.65 (0.11)	4.94	71	< .001	0.70	0.39—1.02
*Confidence (correct answers)*							
Sample 1	3.74 (0.72)	4.64 (0.60)	10.92	69	< .001	1.34	1.01—1.68
Sample 2	3.52 (0.94)	4.72 (0.66)	8.38	37	< .001	1.44	0.95—1.93
Sample 3	4.06 (0.65)	4.88 (0.59)	8.25	36	< .001	1.32	0.88—1.75
Sample 4	4.07 (0.65)	4.60 (0.62)	5.35	36	< .001	0.84	0.47—1.20
Sample 5	4.11 (0.72)	4.93 (0.66)	10.7	71	< .001	1.20	0.91—1.49
*Confidence (incorrect answers)*							
Sample 1	2.73 (0.74)	3.33 (0.96)	6.16	69	< .001	0.69	0.44—0.93
Sample 2	2.58 (1.04)	3.56 (1.04)	6.50	37	< .001	0.95	0.60—1.30
Sample 3	3.67 (0.77)	4.35 (0.85)	5.25	36	< .001	0.84	0.47—1.21
Sample 4	3.70 (0.66)	4.12 (0.75)	3.57	36	< .001	0.60	0.23—0.96
Sample 5	3.57 (0.80)	4.34 (0.90)	8.05	71	< .001	0.91	0.64—1.18

### Confidence distributions

**Fig. 3:**
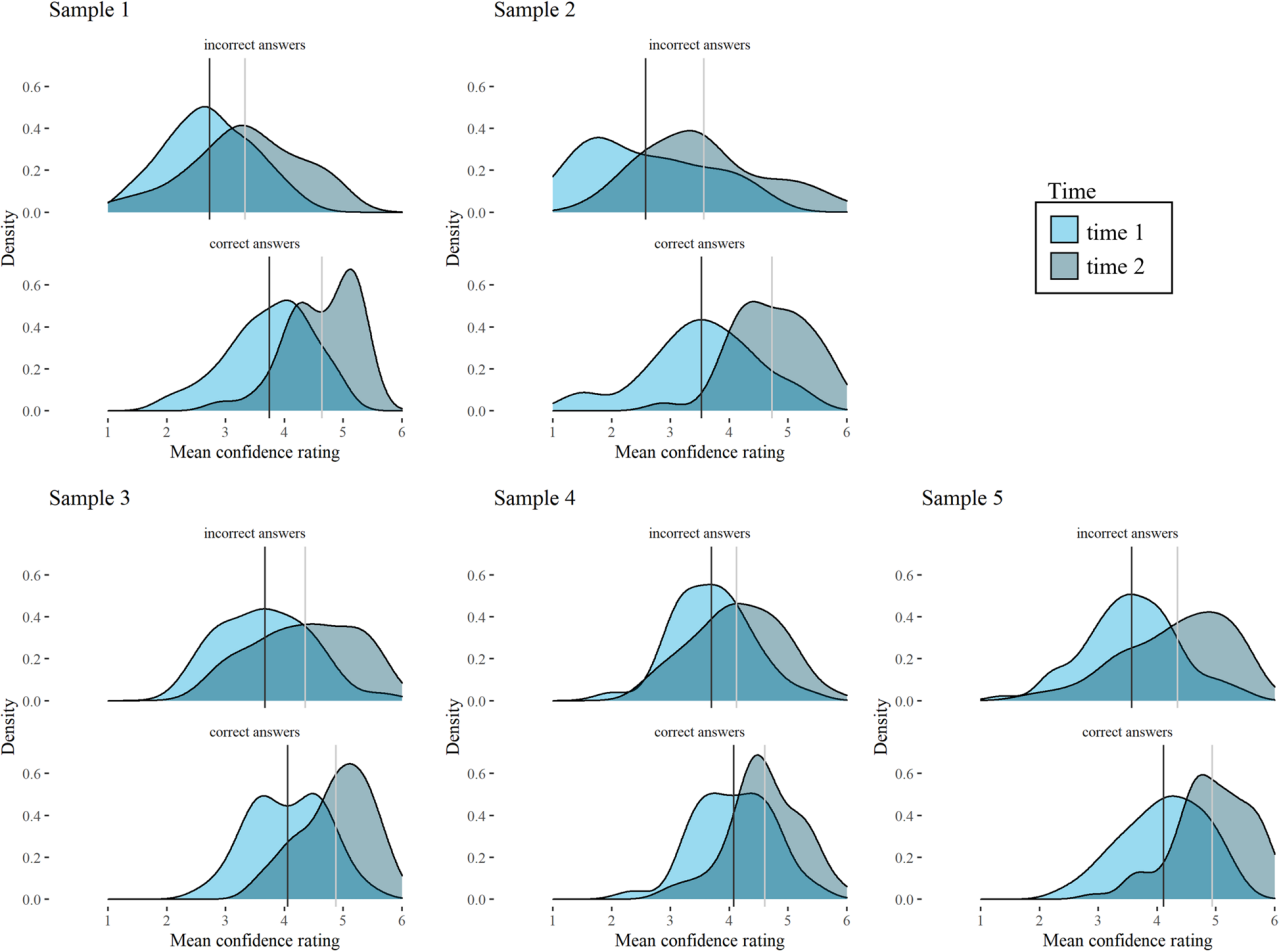
Distributions of mean confidence ratings at time 1 and time 2 for all samples, separated for correct and incorrect answers. Vertical lines indicate means of distributions

Figure 3 shows distributions of confidence at t1 and t2 for all samples separated for correct and incorrect answers. After course units, distributions for correct answers were steeper and had a longer left tail at t2, indicating an increase in high confidence responses or an increase in knowing what is known. For incorrectly answered questions, this pattern was similar, although curves were flatter overall. The distributions shifted to the right at t2, the opposite of what would be expected if individuals improved upon knowing what they do not know.

### Change in confidence over time

**Fig. 4:**
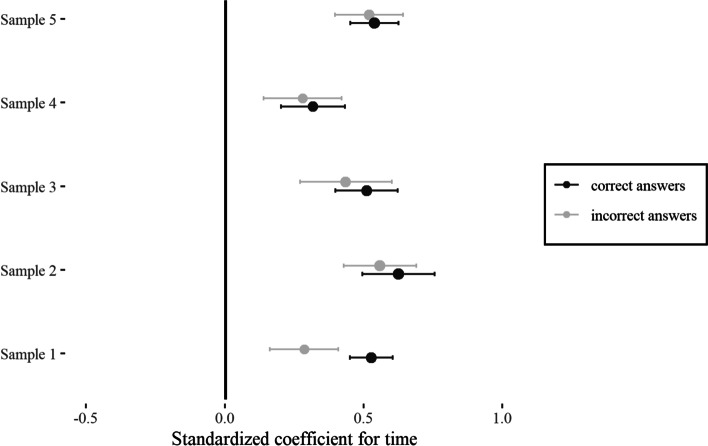
Standardized mixed effects regression coefficients for time separated for sample and correct/incorrect answers including confidence intervals

For answering our main research question of whether students would develop false certainty after one course unit, we fitted several mixed effects models to data containing confidence responses to the knowledge test questions. Figure 4 shows the standardized mixed effects regression coefficients of the predictor time for all samples, separated for correct/incorrect answers. All coefficients showed a similar pattern: Confidence intervals did not cross zero, indicating that confidence increased significantly in all samples. The mean effect size of confidence increase over time aggregated over all samples was *M* = 0.50 (*SD* = 0.11) for correct questions and *M* = 0.41 (*SD* = 0.12) for incorrect ones. The latter finding supports the hypothesis that false certainty already developed after one course unit. See supplementary material for statistical parameters of all analyses (Supplement A).

### Relative metacognitive accuracy

To answer the research question of how metacognitive calibration changes after learning, we analysed mean relative metacognitive accuracy scores by conducting paired sample t-tests for scores at t1 and t2 for each sample. The results showed a different pattern for Samples 1 and 2 than for Samples 3, 4, and 5. Sample 1 showed low relative metacognitive accuracy at t1 (*M* = 0.36, *SD* = 0.31) but participants were able to improve to a moderate degree at t2 (*M* = 0.52, *SD* = 0.24; *d* = 0.46; *p* = 0.005). Sample 2 displayed higher relative metacognitive accuracy at t1 (*M* = 0.45, *SD* = 0.28) but did not improve significantly (*p* = 0.403). Students of Samples 3–5 had low relative metacognitive accuracy at t1 and showed improvement at t2. However, only the increase of Sample 5 from *M* = 0.28 (*SD* = 0.31) to *M* = 0.40 (*SD* = 0.35) reached statistical significance (*p* = 0.003). Table 3 shows the statistical parameters of analyses conducted including confidence intervals for effect sizes.

**Table 3:** Statistical parameters for changes of mean relative metacognitive accuracy including effect sizes and their confidence intervals

	**t1**	**t2**					
**Sample**	**M (SD)**	**M (SD)**	**t**	**df**	**p**	**Cohen’s_d**	**CIs**
Sample 1	0.42 (0.27)	0.54 (0.26)	2.92	69	.005	0.46	0.13—0.78
Sample 2	0.40 (0.32)	0.45 (0.37)	0.85	37	.403	0.15	-0.21—0.51
Sample 3	0.20 (0.39)	0.30 (0.35)	1.21	33	.234	0.27	-0.18—0.73
Sample 4	0.21 (0.32)	0.29 (0.31)	1.26	32	.216	0.25	-0.15—0.65
Sample 5	0.27 (0.32)	0.40 (0.34)	3.13	65	.003	0.39	0.13—0.64

## Discussion

This study was conducted to examine the changes in medical and physiotherapy students’ confidence in their knowledge and metacognitive calibration after only one course unit in different educational contexts. The data showed substantial differences in mean confidence scores and discrimination ability, illustrating a complex picture of students’ metacognition. In general, results revealed that students’ metacognitive calibration could reach a moderate level after learning, although it was not often the case and far from optimal: Only students in two out of five samples were able to improve their discrimination ability. Most importantly, we demonstrated a robust substantial increase in false certainty, that is, increased confidence in incorrect answers to knowledge test items across all samples. Since measures of calibration are derived from confidence ratings and their association, increased false certainty might be responsible for the missed opportunity to improve discrimination ability after learning.

Before and after course units, all students did on average report higher confidence in correctly than in incorrectly answered questions, which is a typical finding [33] and mirrors the above-zero discrimination ability captured with our metacognitive calibration measure. Taken together, the results show that students were capable of moderate discrimination between their correct and incorrect answers. But there was substantial variation among different cohorts and across different course units regarding pre-existing discrimination ability and improvement after learning.

The results for general confidence measured separately for correct and incorrect answers were robust across samples: Confidence in correct answers increased after learning, but also confidence in incorrect ones. This effect of increased false certainty had a medium effect size and was nearly as large as the effect of the increase of confidence in correct answers. This could mean that after a learning experience, although students are able to better judge what they know, they also display false certainty—hence are worse in knowing what they do not know. Similar findings have been reported for short online information search scenarios [34]. Also, another recent study reports that more knowledgeable students are able to give a high confidence response more adequately than low performing students when they are actually correct [35]. However, they are worse in applying the low confidence response to wrong questions. Our findings complement findings of longitudinal studies that showed increased confidence over the longer course of academic education [17, 22]. To our knowledge this study is the first to illustrate that in health professional education one course unit alone can elicit false certainty. Ongoing learning experiences, where students acquire more knowledge, accompanied by increased false certainty, could accumulate over the course of a student’s medical education and lead to the effects observed in longitudinal studies.

Knowing what one knows and what one does not know is a highly important learning goal for health professionals because it is part of critically reflecting upon one’s own clinical decisions [36]. Having high confidence in wrong knowledge is potentially hazardous in clinical practice [37]. This means seeing increasing false certainty developing after only one course unit is troubling for educators in health professional education. Our results also support what has already been pointed out by other researchers: Medical professionals can be reluctant to admit their uncertainty in medical diagnoses [38], and students need to be familiarized with experiencing uncertainty [39, 40] and the feeling of not knowing something [1, 13] in their future work life. One limitation of our study is that it cannot be ruled out that initially emerging overall high confidence could fade away after subsequent learning experiences in additional course units. For probabilistic learning tasks, studies showed that learners can find themselves in a “beginner’s bubble” of overconfidence marked by quickly developing overconfidence after a little learning, which gives way to better metacognitive calibration after more learning [41, 42]. It has, however, also been shown that confidence in one’s ability after a short learning period may be more stable than knowledge itself [43], which points to a potential resilience of false certainty once it has emerged. Future research is needed to directly test stability of false certainty in health professional education. Another limitation concerns the generalizability of our results to different topics in health professional education. Our study investigated confidence change after learning only for knowledge in anatomy and gait analysis. Although similar confidence effects have been demonstrated for topics like meteorology [44], scuba diving [34], and overall clinical knowledge [17], to infer about its generalizability, the causes of this effect have yet to be investigated.

## Conclusions

Our results alert educators that students of health professions are potentially capable of moderate metacognitive calibration but at the same increase their confidence in incorrect answers after only one course unit. This developing false certainty is troubling as high confidence in wrong medical knowledge, if persistent in professional life, can ultimately threaten patient safety. Medical and physiotherapy educators should be aware of this effect and think of means to counter it. Students need to be made familiar with uncertainty and not knowing, since these are part of most clinical routines. This could help to reduce diagnostic error in future professional practice and improve overall metacognition of health professionals.

## Supplementary Information

**Additional file 1: **Supplementary material.

## Data Availability

Data and code for analysis is provided at https://osf.io/6v8za/?view_only=4a8fd507cceb42c1b8a8e9070d247728.
