# Supplementary material for: False certainty in the acquisition of anatomical and physiotherapeutic knowledge
Source: BMC Med Educ. 2022 Nov 8;22:765. doi: 10.1186/s12909-022-03820-x (PMC9641864; doi:10.1186/s12909-022-03820-x)
Supplement: Supplementary file 1 — Additional file 1: Supplementary material. [file 12909_2022_3820_MOESM1_ESM.docx]

**False certainty in the acquisition of medical knowledge - Supplementary material**

## A: Results of mixed effects regressions

For all models, regression estimates for fixed effects were standardized by z-transformation of the confidence variable. Id denotes the identification number of each participant. Item refers to each item of the knowledge test. Random effects include residual variance (σ2) as well as variances for id (τ0 id), id * time slope (τ1 id * time), and random intercept for item (τ2 item). Time was coded 0 = time 1 and 1 = time 2.

| *Table 1:* Mixed effects regressions of time on z-transformed variable confidence for incorrect and correct answers for Sample 1. | | | | | | |
| --- | --- | --- | --- | --- | --- | --- |
|  | **confidence in incorrect answers** | | | **confidence in correct answers** | | |
| *Predictors* | *Estimates* | *CI* | *p* | *Estimates* | *CI* | *p* |
| (Intercept) | -0.11 | -0.36 – 0.14 | 0.387 | -0.41 | -0.65 – -0.17 | 0.001 |
| time (t2) | 0.29 | 0.16 – 0.41 | **<0.001** | 0.53 | 0.45 – 0.60 | **<0.001** |
| **Random Effects** |  |  |  |  |  |  |
| σ^2^ | 0.52 |  |  | 0.52 |  |  |
| τ_0_ _id_ | 0.12 |  |  | 0.1 |  |  |
| τ_1_ _id * time_ | 0.11 |  |  | 0.04 |  |  |
| τ_2_ _item_ | 0.3 |  |  | 0.31 |  |  |
| ICC | 0.45 |  |  | 0.45 |  |  |
| N _id_ | 70 |  |  | 70 |  |  |
| N _item_ | 24 |  |  | 24 |  |  |
| Observations | 1066 |  |  | 2273 |  |  |
| Marginal R^2^ Conditional R^2^ | 0.021 0.461 |  |  | 0.069 0.485 |  |  |
|  | | | | | | |
| *Table 2:* Mixed effects regressions of time on z-transformed variable confidence for incorrect and correct answers for Sample 2. | | | | | | |
|  | **confidence in incorrect answers** | | | **confidence in correct answers** | | |
| *Predictors* | *Estimates* | *CI* | *p* | *Estimates* | *CI* | *p* |
| (Intercept) | -0.18 | -0.45 – 0.10 | 0.001 | -0.49 | -0.78 – -0.19 | 0.001 |
| time (t2) | 0.56 | 0.43 – 0.69 | **<0.001** | 0.63 | 0.50 – 0.75 | **<0.001** |
| **Random Effects** |  |  |  |  |  |  |
| σ^2^ | 0.54 |  |  | 0.41 |  |  |
| τ_0_ _id_ | 0.21 |  |  | 0.16 |  |  |
| τ_1_ _id * time_ | 0 |  |  | 0.11 |  |  |
| τ_2_ _item_ | 0.26 |  |  | 0.44 |  |  |
| ICC | - |  |  | 0.6 |  |  |
| N _id_ | 38 |  |  | 38 |  |  |
| N _item_ | 24 |  |  | 24 |  |  |
| Observations | 545 |  |  | 1267 |  |  |
| Marginal R^2^ Conditional R^2^ | 0.126 - |  |  | 0.089 0.632 |  |  |
|  |  |  |  |  |  |  |
| *Table 3:* Mixed effects regressions of time on z-transformed variable confidence for incorrect and correct answers for Sample 3. | | | | | | |
|  | **confidence in incorrect answers** | | | **confidence in correct answers** | | |
| *Predictors* | *Estimates* | *CI* | *p* | *Estimates* | *CI* | *p* |
| (Intercept) | -0.16 | -0.37 – 0.04 | 0.115 | -0.41 | -0.64 – -0.18 | 0.001 |
| time (t2) | 0.43 | 0.27 – 0.60 | **<0.001** | 0.51 | 0.40 – 0.62 | **<0.001** |
| **Random Effects** |  |  |  |  |  |  |
| σ^2^ | 0.69 |  |  | 0.62 |  |  |
| τ_0_ _id_ | 0.16 |  |  | 0.09 |  |  |
| τ_1_ _id * time_ | 0.08 |  |  | 0 |  |  |
| τ_2_ _item_ | 0.08 |  |  | 0.19 |  |  |
| ICC | 0.27 |  |  | - |  |  |
| N _id_ | 37 |  |  | 37 |  |  |
| N _item_ | 20 |  |  | 20 |  |  |
| Observations | 647 |  |  | 791 |  |  |
| Marginal R^2^ Conditional R^2^ | 0.048 0.301 |  |  | 0.095  - |  |  |
|  |  |  |  |  |  |  |
| *Table 4:* Mixed effects regressions of time on z-transformed variable confidence for incorrect and correct answers for Sample 4. | | | | | | |
|  | **confidence in incorrect answers** | | | **confidence in correct answers** | | |
| *Predictors* | *Estimates* | *CI* | *p* | *Estimates* | *CI* | *p* |
| (Intercept) | -0.14 | -0.36 – 0.08 | 0.216 | -0.33 | -0.59 – -0.07 | 0.012 |
| time (t2) | 0.28 | 0.14 – 0.42 | **<0.001** | 0.32 | 0.20 – 0.43 | **<0.001** |
| **Random Effects** |  |  |  |  |  |  |
| σ^2^ | 0.71 |  |  | 0.56 |  |  |
| τ_0_ _id_ | 0.12 |  |  | 0.09 |  |  |
| τ_1_ _id * time_ | 0.02 |  |  | 0.02 |  |  |
| τ_2_ _item_ | 0.14 |  |  | 0.26 |  |  |
| ICC | 0.26 |  |  | 0.39 |  |  |
| N _id_ | 37 |  |  | 37 |  |  |
| N _item_ | 20 |  |  | 20 |  |  |
| Observations | 670 |  |  | 789 |  |  |
| Marginal R^2^ Conditional R^2^ | 0.020 0.278 |  |  | 0.027 0.406 |  |  |
|  |  |  |  |  |  |  |
| *Table 5:* Mixed effects regressions of time on z-transformed variable confidence for incorrect and correct answers for Sample 5. | | | | | | |
|  | **confidence in incorrect answers** | | | **confidence in correct answers** | | |
| *Predictors* | *Estimates* | *CI* | *p* | *Estimates* | *CI* | *p* |
| (Intercept) | -0.23 | -0.41 – -0.05 | 0.013 | -0.44 | -0.67 – -0.22 | <0.001 |
| time (t2) | 0.52 | 0.40 – 0.64 | **<0.001** | 0.54 | 0.45 – 0.63 | **<0.001** |
| **Random Effects** |  |  |  |  |  |  |
| σ^2^ | 0.62 |  |  | 0.57 |  |  |
| τ_0_ _id_ | 0.18 |  |  | 0.12 |  |  |
| τ_1_ _id * time_ | 0.1 |  |  | 0.04 |  |  |
| τ_2_ _item_ | 0.09 |  |  | 0.22 |  |  |
| ICC | 0.3 |  |  | 0.37 |  |  |
| N _id_ | 72 |  |  | 72 |  |  |
| N _item_ | 19 |  |  | 20 |  |  |
| Observations | 1112 |  |  | 1738 |  |  |
| Marginal R^2^ Conditional R^2^ | 0.070 0.352 |  |  | 0.074 0.419 |  |  |

| *Table 6:* Betas, confidence intervals and number of observations for confidence in incorrect and correct answers for all samples. | | | | | | |
| --- | --- | --- | --- | --- | --- | --- |
| *Sample* | *beta* | *CIs* | *N_observations_* | *beta* | *CIs* | *N_observations_* |
| **incorrect answers** | | | | **correct answers** | | |
| Sample 1 | 0.29 | 0.16 - 0.41 | 1066 | 0.53 | 0.45 - 0.60 | 2273 |
| Sample 2 | 0.56 | 0.43 - 0.69 | 545 | 0.63 | 0.50 - 0.76 | 1267 |
| Sample 3 | 0.43 | 0.27 - 0.60 | 647 | 0.51 | 0.40 - 0.62 | 791 |
| Sample 4 | 0.28 | 0.14 - 0.42 | 670 | 0.32 | 0.20 - 0.43 | 789 |
| Sample 5 | 0.52 | 0.40 - 0.64 | 1112 | 0.54 | 0.45 - 0.63 | 1738 |
| Mean | **0.42** |  |  | **0.51** |  |  |
| SD | **0.12** |  |  | **0.10** |  |  |
